# Video Endoscopy-Guided Intrabronchial Spray Inoculation of *Mycobacterium bovis* in Goats and Comparative Assessment of Lung Lesions With Various Imaging Methods

**DOI:** 10.3389/fvets.2022.877322

**Published:** 2022-05-03

**Authors:** Nadine Wedlich, Julia Figl, Elisabeth M. Liebler-Tenorio, Heike Köhler, Kerstin von Pückler, Melanie Rissmann, Stefanie Petow, Stefanie A. Barth, Petra Reinhold, Reiner Ulrich, Leander Grode, Stefan H. E. Kaufmann, Christian Menge

**Affiliations:** ^1^Institute of Molecular Pathogenesis, Friedrich-Loeffler-Institute (FLI), Jena, Germany; ^2^Clinic for Small Animals – Radiology, Justus-Liebig-University Giessen, Giessen, Germany; ^3^Department of Experimental Animal Facilities and Biorisk Management, Friedrich-Loeffler-Institute, Greifswald-Insel Riems, Germany; ^4^Institute for Animal Welfare and Animal Husbandry, Friedrich-Loeffler-Institute, Celle, Germany; ^5^Vakzine Projekt Management GmbH, Hannover, Germany; ^6^Director Emeritus, Max Planck Institute for Infection Biology, Berlin, Germany; ^7^Emeritus Group for Systems Immunology, Max Planck Institute for Multidisciplinary Sciences, Göttingen, Germany; ^8^Hagler Institute for Advanced Study, Texas A&M University, College Station, TX, United States

**Keywords:** tuberculosis, *Mycobacterium bovis*, goat, inoculation, spray catheter, imaging, CT, histology

## Abstract

Bovine tuberculosis (bTB) not only poses a zoonotic threat to humans but also has a significant economic impact on livestock production in many areas of the world. Effective vaccines for humans, livestock, and wildlife are highly desirable to control tuberculosis. Suitable large animal models are indispensable for meaningful assessment of vaccine candidates. Here, we describe the refinement of an animal model for bTB in goats. Intrabronchial inoculation procedure *via* video-guided endoscopy in anesthetized animals, collection of lungs after intratracheal fixation *in situ*, and imaging of lungs by computed tomography (CT) were established in three goats using barium sulfate as surrogate inoculum. For subsequent infection experiments, four goats were infected with 4.7 × 10^2^ colony-forming units of *M. bovis* by intrabronchial inoculation using video-guided endoscopy with spray catheters. Defined amounts of inoculum were deposited at five sites per lung. Four age-matched goats were mock-inoculated. None of the goats developed clinical signs until they were euthanized 5 months post infection, but simultaneous skin testing confirmed bTB infection in all goats inoculated with *M. bovis*. In tissues collected at necropsy, *M. bovis* was consistently re-isolated from granulomas in lymph nodes, draining the lungs of all the goats infected with *M. bovis*. Further dissemination was observed in one goat only. Pulmonary lesions were quantified by CT and digital 2D radiography (DR). CT revealed mineralized lesions in all the infected goats ranging from <5 mm to >10 mm in diameter. Small lesions <5 mm predominated. The DR failed to detect small lesions and to determine the exact location of lesions because of overlapping of pulmonary lobes. Relative volume of pulmonary lesions was low in three but high in one goat that also had extensive cavitation. CT lesions could be correlated to gross pathologic findings and histologic granuloma types in representative pulmonary lobes. In conclusion, video-guided intrabronchial inoculation with spray catheters, mimicking the natural way of infection, resulted in pulmonary infection of goats with *M. bovis*. CT, but not DR, presented as a highly sensitive method to quantify the extent of pulmonary lesions. This goat model of TB may serve as a model for testing TB vaccine efficacy.

## Introduction

Infections of ruminants with *M. bovis* and its close relative *M. caprae* have a significant economic impact on livestock production in many areas of the world (1–3), threaten wildlife populations (4), and cause zoonotic infections (5). Human tuberculosis (1) is predominantly associated with *Mycobacterium* (*M*.) *tuberculosis* infections and caused an estimated 1.5 million deaths in 2020 (4). Preventive measures in human medicine still mostly rely on vaccination with *Bacille Calmette-Guérin* (BCG), a vaccine developed almost a century ago. Intense efforts are ongoing to develop improved vaccines, specifically designed for certain age groups, individuals with co-infections, or distinct routes of application (6–8).

Appropriate, standardized animal models are indispensable to evaluate and compare the efficacy of new vaccines and immunization regimens. Information derived from laboratory rodent models allow for only limited translational extrapolation because of differences in respiratory anatomy and physiology, as well as immunologic features (9–11). Optimal non-human primate models have limited availability, as well as significant ethical and financial constraints (12). Goats are a well-suited model species for mycobacterial infections in both humans and ruminants (6, 13–15). The anatomy of their respiratory tract resembles more closely that of humans than that of rodents with regard to complex branching airways (16), vascularization (5, 17), and distribution of mucosa-associated lymphoid tissues (18). The size and body weight of goats are in the range of those of humans. This is advantageous, since the diameter of airways correlates with body weight (16). Goats are susceptible to natural infection with *M. bovis* and *M. caprae* and develop tuberculosis (19, 20). The disease takes a chronic course resulting in pulmonary lesions comparable to those in humans, e.g., granulomas and caverns (14, 20). Tuberculous granulomas in goats are fibrotic like in human TB which impedes the penetration of anti-TB drugs and efficacy of chemotherapy. This is difficult to reproduce in other animal models (21).

Goat models, however, are more difficult to standardize compared to laboratory rodent models, since goats are an out-bread species resulting in higher individual variation. High numbers of experimental animals, on the other hand, are generally not feasible for practical reasons. Therefore, it is important that variables, like route of inoculation and read-out parameters, are standardized. Inoculation must be practicable, reproducible, and safe in terms of both unwanted adverse effects in the animals hampering the development of natural disease progression, and work safety for the experimenter. Different routes of inoculation, e.g., intratracheal, transthoracic, and endobronchial, using low doses of *M. bovis* or *M. caprae* have been successfully used (13, 15, 22, 23). These applications have in common that bacterial suspensions are administered as a bolus. The most common source of natural TB infection, however, are aerosolized bacteria which are inhaled and deposited in the respiratory tract of the recipient (24). This prompted the establishment of a caprine aerosol *M. bovis* infection model (21). Unfortunately, the model required high inoculation doses and did not result in the formation of cavernous lung lesions. Frequent development of such lesions is one of the peculiarities observed in caprine TB infection studies and an additional advantage of goats as human TB models. The seeder animal approach, e.g., exposure to infected goats by close contact, mimics natural infection most closely and is effective (25, 26). However, it suffers from the significant disadvantage that the exact time of infection and the infectious dose for individual animals remain unknown. High-definition video endoscopy and application *via* spray catheter to ensure optimal distribution have been used for other purposes, e.g., to distribute dyes throughout the colon resulting in higher detection rate of intraepithelial neoplasia in clinical oncology (27). Spray catheters have also been suggested as devices for intrathoracic aerosol chemotherapy (28). The use of spray catheters in respiratory infection studies would allow for aerosolizing the inoculum at defined sites in the bronchial tree while, at the same time, avoiding the disadvantages of aerosol application.

Current anti-TB vaccines only ameliorate lesion formation and bacterial load (29), making quantitative evaluation of lesions at high resolution indispensable to reliably unveil differences in vaccine efficacy. For large animal TB models, slicing of the entire lungs and semiquantitative scoring are commonly used, but imaging technologies like radiography (30), magnetic resonance imaging (31), and computed tomography (32) have also been assessed (13, 33, 34). A comparative evaluation of semiquantitative scoring of thinly sliced lungs and CT demonstrated superior quality of CT (13) and the latter technique was successfully applied in a series of TB studies on goats (23, 35). A limitation of this approach is, however, that the lungs have to be removed from infected animals, conserved, and treated to be no longer infectious, as CT devices are not regularly available in high containment animal facilities. This prevents the use of CT to monitor the development of TB lesions *intra vitam* during the course of the experiment. This could be more easily achieved by classical or digital 2D radiography (DR).

The aim of this study was further refinement of the infection model for *M. bovis* in goats. The endoscopy-guided deposition of a suspension at defined sites of the respiratory tract of young goats was established. Distribution of the inoculum in the lungs was investigated, and parameters for pulmonary CT analysis were defined. Lungs were fixed *in situ* to retain the original size of the lungs, thereby avoiding artifacts and leveraging objective determination of lesion sizes. The protocol applied for inoculation with *M. bovis via* spray catheter was used for this purpose for the first time to the best of our knowledge. Number, size, and spatial distribution of pulmonary lesions were assessed by CT imaging and digital radiography, and were compared to macroscopic and histological findings.

## Materials and Methods

### Establishment of Intrabronchial Application Procedure and Pulmonary CT Imaging (Trial 1)

#### Animals

Three male castrated goats of the breed German Improved White were purchased from a conventional farm. They were transferred at the age of 3 months to the animal facility at Friedrich-Loeffler-Institut, Jena, Germany (FLI Jena). The goats were kept as one group on straw bedding, had access to hay and water *ad libitum*, and received age-adjusted quantities of concentrated feed. They had been vaccinated against *Clostridium* spp., *Mannheimia haemolytica*, and *Pasteurella trehalosi* in their herd of origin. Upon arrival at FLI Jena, they were screened for bacterial infections with *Salmonella* spp., *Mycoplasma* spp., *Pasteurella* spp., *Mycobacterium* spp., *Coxiella burnetti*, and *Chlamydia* spp. as well as for ecto- and endoparasites. They were negative for bacterial infections, but all the goats were infected with *Eimeria* spp. They were treated with antibiotics for 5 days [Baytril 10%; Bayer, Leverkusen, Germany; 2.5 mg/kg body weight (BW), s.c.] as a preventive measure and with a single dose of antiparasitics (Baycox; Bayer; 20 mg/kg BW, oral) within the first 2 weeks at FLI Jena. They were examined daily for general behavior, appetite, clinical symptoms, and rectal temperature. Their health status was documented using a scoring system.

#### Legislation and Ethical Approval

This study was carried out in strict accordance with European and National Law for the Care and Use of Animals. The protocol was reviewed by the Committee on the Ethics of Animal Experiments of the State of Thuringia, Germany, and approved by the competent authority (permit number: 22-2684-04-04-101/16, date of permission 20.04.2016). Every effort was made to minimize the suffering of the animals during the entire study. The stables were equipped with enrichments for activity, e.g., chains and different equipment for climbing.

#### Intrabronchial Application of Barium Sulfate

The goats were healthy, notably free of respiratory disease, and weighed ~30 kg at the time of intrabronchial application. The animals were sedated with midazolam (Midazolam-ratiopharm^©^ 15 mg/ml; Ratiopharm, Ulm, Germany; 0.5 mg/kg BW) and ketamine (Ketamin 10%, WDT, Garbsen, Germany; 10 mg/kg BW) intramuscularly. Then, they were transferred to an examination room and anesthetized intravenously (IV) with midazolam (Midazolam-ratiopharm^©^ 15 mg/ml; 0.1 mg/kg BW) and ketamine (Ketamin 10%; 5 mg/kg BW). The pharynx was locally anesthetized by spraying with 2% lidocaine (Betapharm Arzneimittel, Augsburg, Germany).

**Figure 1:**
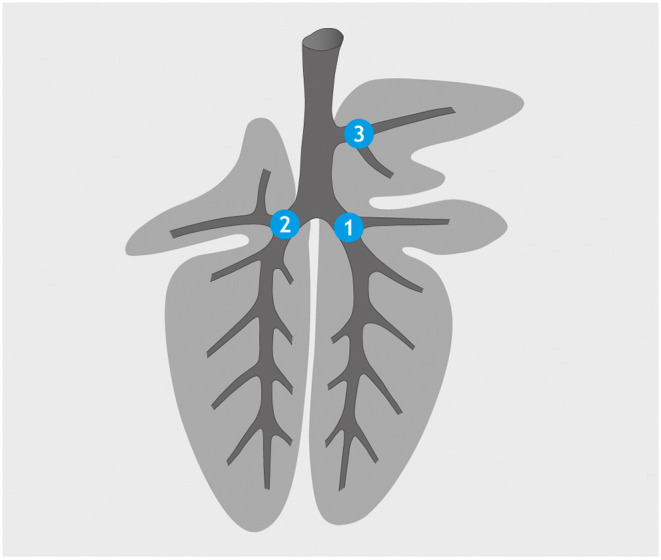
Application sites for barium sulfate suspension (standard catheter): *bronchus* (*b*.) *principalis dexter* (1), *b. principalis sinister* (2), and *b. tracheobronchialis* (3).

For guided intrabronchial application of liquids, a technique described by Prohl et al. (36) for calves was adapted. The anesthetized goats were positioned in sternal recumbency on an examination table. In this trial, the 5.2 mm diameter of the bronchoscope was too big for the nasal passage of the goats and had to be inserted orally. A plastic tube (diameter: 2 cm, length: 20 cm) was placed into the goat's mouth, and a flexible video endoscope of 85 cm working length and outer diameter of 5.2 mm (Veterinary Video Endoscope 60001 VL 1, Storz, Tuttlingen, Germany) was inserted through the mouth into the trachea. To be able to immediately control the distribution of the inocula, a barium sulfate suspension (1 g BaSO_4_ in 2.5 ml H_2_O; WDT, Garbsen, Germany) was applied as radiography dense reference liquid. Aliquots of 1 ml of the barium sulfate suspension were placed at the *bronchus (b.) tracheobronchialis, b. principalis dexter*, and *b. principalis sinister* (Figure 1). Because of its viscosity, the suspension was applied through a standard catheter.

#### Necropsy

The goats were euthanized with pentobarbital-sodium (Release^©^, WDT; 150 mg/kg BW, IV) immediately after intrabronchial application while still in deep anesthesia. The lungs were fixed by intratracheal instillation with 4% neutral buffered formalin (NBF). For this, the goats were positioned on their back. The trachea was exposed and cut caudal of the larynx. A flexible tube attached to a container with 4% NBF located 30 cm above the thorax (for a pressure of about 30 mbar) was inserted into the trachea and 1.5 l of fixative allowed to fill the lungs. After 15 min, the trachea was closed, the thoracic cavity was opened, and the lungs were carefully removed. Mediastinal lymph nodes, the heart, and the esophagus were detached. The pulmonary surface was visually inspected, and gross lesions were documented. The lungs were stored in 4% NBF at 4°C.

#### Radiological Examination by Computed Tomography

For CT, the lungs were removed after 2 weeks from NBF and placed into two plastic bags inside a plastic container. The optimal settings for scanning were evaluated in this trial. The lungs were scanned in a position corresponding to sternal recumbency in a computer tomograph (Brilliance 16; Phillips, Hamburg, Germany) at 120 kV and 0.8 mm slice thickness and the use of the lung algorithm. Virtual slices were analyzed with Synedra personal view^©^ (www.synedra.com). Initially, the lungs were scanned in plastic bags with a minute volume of formalin around the tissue. Since the intrapulmonary fixative obscured the airways, liquids were drained as much as possible from the lungs in the following scans.

### Inoculation of Goats With *M. bovis, intra vitam*, and *post mortem* Studies (Trial 2)

#### Animals

For the infection experiment, eight male goats were obtained from the same source as in trial 1 and transferred to FLI Jena at an average age of 24 days. The nutrition regime was changed from milk replacer to a diet with concentrated feed, hay or hay cobs, and water *ad libitum* during the 1st weeks of life. The goats were kept in two groups in separate rooms on straw bedding. They had been vaccinated, were screened for infections, and received treatment as described for trial 1. They were castrated at the age of 3 months under sedation and local as well as systemic analgesia using a Burdizzoe emasculatome. At the age of 6 months, the goats were transferred to the biosafety level three animal facility at FLI in Greifswald–Insel Riems, Germany (FLI Riems). Air-conditioned rooms were located in the center of an experimental animal facility with several layers of negative air-pressure to prevent any release of pathogens. Rooms used to keep the animals for this study had an overall negative air pressure of 120 Pa relative to the outside environment and an air exchange rate of 9 times/h. The outgoing air was passed through HEPA filters. The rooms had artificial light for 12 h a day and were equipped with rubber mattresses. Chains, platforms for climbing, branches, and balls served as enrichment as described for trial 1.

#### Legislation and Ethical Approval

This study was carried out in strict accordance with European and National Law for the Care and Use of Animals. The protocol was reviewed by the Committee on the Ethics of Animal Experiments of the State of Mecklenburg-Western Pomerania, Germany and approved by the competent authority (permit number: 7221.3-1-084/16, date of permission: 08.02.2017). The infection experiment was conducted in a contained area at biosafety level 3 under supervision of the authorized institutional Agent for Animal Protection. Bronchoscopy was strictly performed under general anesthesia as described above. During the entire study, every effort was made to minimize the suffering of the animals.

#### Inoculum

Strain *M. bovis* SB0989 [collection of the German National Reference Laboratory for Bovine Tuberculosis, FLI Jena (37)] was cultured in Middlebrook 7H9 (MB) bouillon for 4 months, characterized by real-time PCR targeting IS*1081* (38), and colony count was determined. The bacterial suspension was cryo-conserved in MB bouillon supplemented with glycerin and an antibiotic mixture containing polymyxin B, amphotericin B, nalidixic acid, trimethoprim, and azlocillin, and stored at −80°C. For inoculation, the strain was defrosted and transferred into phosphate-buffered saline (PBS). In brief, the suspension was centrifuged at 4,000 × *g* for 10 min at 20°C, and the pellet was resuspended in 1 ml sterile PBS. Based on colony counts of the cryo-conserved stock, the inoculation dose was adjusted to achieve ~5 × 10^2^ cfu per animal.

Quantitative re-assessment of the remaining inoculum yielded 4.7 × 10^2^ cfu per animal. In the preceding experiment (data not shown), the colony count of the bacterial suspension before and after passage through a spray catheter had been determined to ascertain that there was no marked loss of bacteria. Quality control of the inoculum after use by endpoint and real-time PCR using the targets IS*1081* (38) and RD4 (32), as well as by spoligotyping (39) and MIRU-VNTR (40, 41) confirmed that the inoculum contained *M. bovis* Lineage BOV_1 SB0989, MIRU-24 profile 222324253322645431223433. However, low-level contamination by *M. caprae* Lineage BOV_4 SB0418, MIRU-24 profile 235424253422435222423421 was discovered. The proportion of the copy number of *M. caprae* in the inoculum amounted to 0.1% of the copy number of *M. bovis* as estimated by quantitative real-time PCR. From the data gathered in this study, particularly the fact that only *M. bovis* was retrieved by culturing samples and tissues from the inoculated animals, we have no reason to suspect that this contamination negatively impacts the interpretation of the data presented.

#### Intrabronchial Inoculation

**Figure 2:**
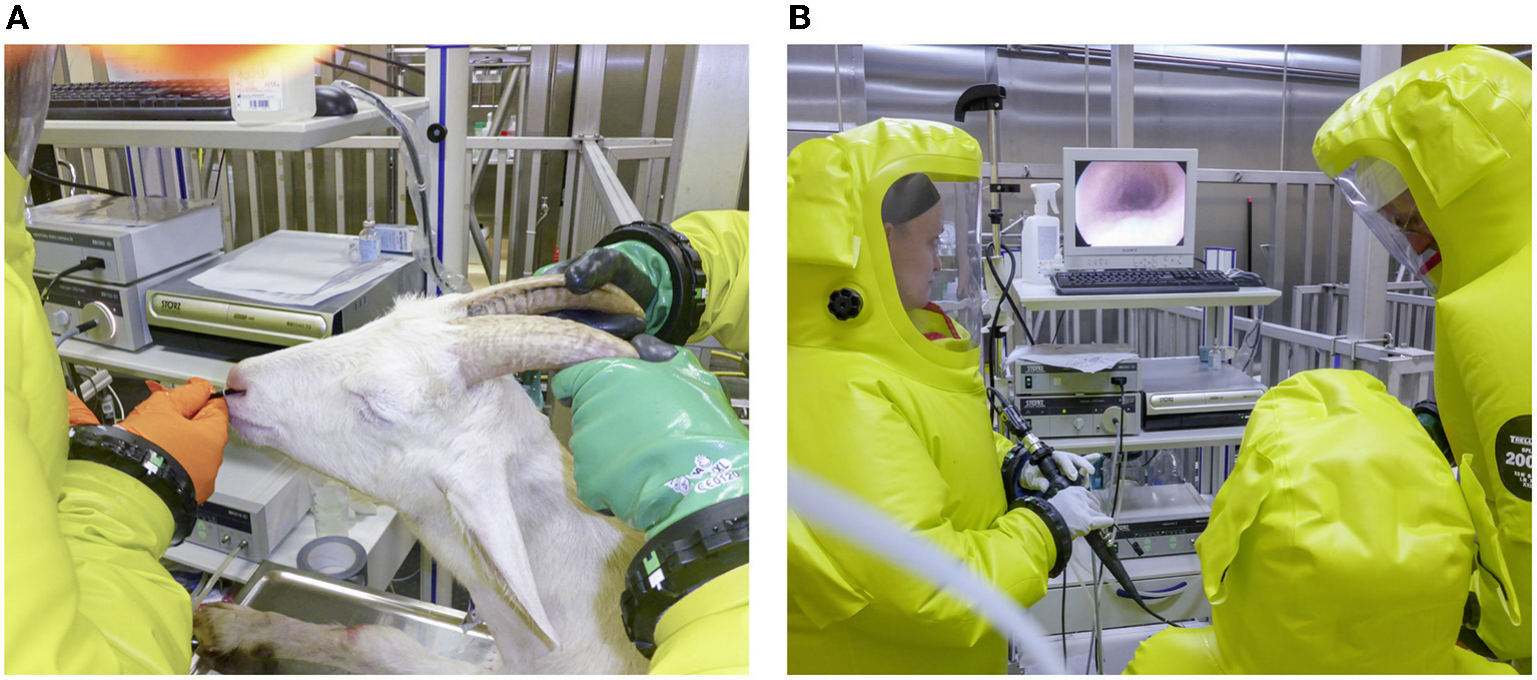
Intrabronchial application of *Mycobacterium bovis* suspension under biosafety level 3 conditions. **(A)** Bronchoscope inserted through the nose of an anesthetized goat in sternal recumbency. **(B)** Bronchoscopic view of the trachea of the goat.

**Figure 3:**
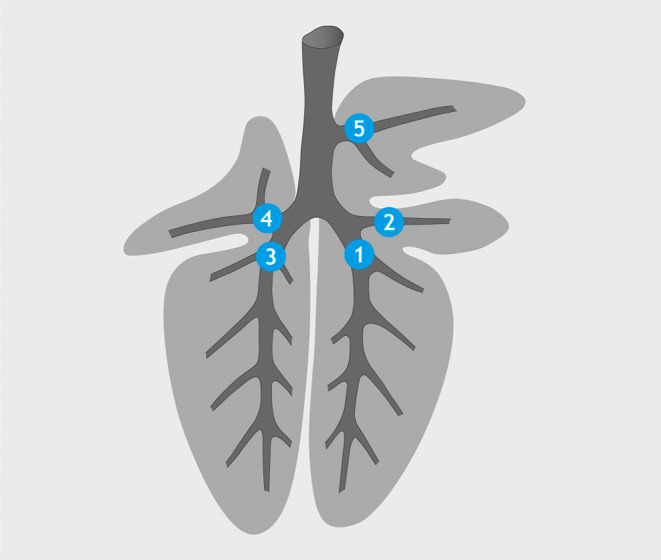
Application sites for *M. bovis* suspension/PBS (spray catheter): *bronchus* (*b*.) *principalis dexter* (1), *b. lobaris medialis* (2), *b. principalis sinister* (3), *b. lobaris cranialis sinister* (4), and *b. tracheobronchialis* (5).

Inoculation was performed as established in trial 1 but using a spray catheter (Storz). Depending on the size of the individual animal (30–50 kg BW), access to the airways was accomplished *via* the ventral nasal passage (Figure 2) or the oral cavity while using a biting protector. Goats in the infection group (*n* = 4) received a 2.5-ml suspension containing 4.7 × 10^2^ cfu of *M. bovis*. Goats in the control group (*n* = 4) were mock-inoculated with 2.5 ml PBS. Aliquots of 500 μl were deposited at five sites, *b. trachealis, b. principales dexter and sinister, b. lobaris cranialis*, and *b. lobaris medialis* (Figure 3). The goats were observed after inoculation until they regained full awareness. Nasal swabs were taken from each goat within 30 min after bronchoscopy. Thereafter, nasal swabs and feces were collected daily for 14 days to monitor a potential excretion of *M. bovis*.

#### Single Intradermal Cervical Comparative Tuberculin (SICCT) Test

SICCT test was performed 137 days post inoculation (dpi, 72 h before necropsy). The goats were injected intradermally with 1 ml of a solution containing either 2,500 IE avian PPD (aPPD; WDT) at the left side and 5,000 IE bovine PPD (bPPD; WDT) at the right side of the neck. Skin fold thickness was measured with a caliper prior and 72 h after injection. Positive response was defined as difference in the increase in skin fold thickness of more than 4 mm between the bPPD and aPPD injection sites, redness or edema at the bPPD injection site, and/or systemic reactions like fever 72 h after injection. Negative response was defined as difference in the increase in skin fold thickness of <2 mm between the bPPD and aPPD injection sites and absence of clinical signs.

#### Necropsy

At the end of the observation period (>140 dpi), the goats were tranquilized with xylacine (Rompun® 2%; Bayer; 0.2 mg/kg BW, IV) followed by euthanasia with pentobarbital-sodium (Release^©^, WDT; 150 mg/kg BW, IV). At first, the lungs were fixed intratracheally as described above followed by complete necropsy. Different tissues were collected for histological examination and cultural isolation of mycobacteria. Sampled tissues were: injection sites of PPDa and PPDb, *lymphonodus* (*LN*) *cervicalis superficialis sinister et dexter, LN mandibularis sinister et dexter, LN parotideus sinister et dexter*, tonsils*, LN retropharyngealis medius sinister et dexter, LN tracheobronchialis sinister, LNN mediastinales*, thymus, heart, liver, spleen, kidney, *LNN renales*, bone marrow, Peyer's patches in jejunum and ileum*, LNN ileocolici*, and *LNN mesenteriales*. Samples for bacterial culture were collected under aseptic conditions. Tissues for histological examination were fixed in 4% NBF. Lungs and tissues for histological examination were stored in the fixative that was renewed after 1 week and after 2 months, and for >2 months at 4°C in the necropsy facility to make sure that *M. bovis* was inactivated.

#### Histology and Ziehl-Neelsen Staining

The right cranial lobe (RCL), right basal lobe (RBL), and accessory lobe (AL) of the lungs of one of the infected goats without superficial lesions and the infected goats with macroscopically visible superficial lesions were cut in 1- (RCL, AL) or 2-cm (RBL) thick slices. Lesions visible on cut surfaces were dissected, and up to five sites per pulmonary lobe were embedded in paraffin. Representative sites, preferentially with macroscopic lesions, of the other sampled tissues were embedded in paraffin.

Paraffin sections were stained with hematoxylin and eosin (H&E) for evaluation of lesions. Granulomas were graded according to a modified scheme from Wangoo et al. (42) (see Supplementary Table 1). Sections from the lungs, tracheobronchial LN, mediastinal LNN, and other sites with granulomas were stained in addition to the Ziehl-Neelsen (ZN) staining protocol for acid-fast bacilli (AFB). AFB numbers were classified as none, single (+, 1 to 10 AFB/section), few (++, 11-50 AFB/section), or many (+++, >50 AFB/section) (43).

#### Microbiological Cultivation

From each tissue sample, 1 g was placed into 10 ml PBS and homogenized with a stomacher device (Interscience, Saint Nom, France) for 6 min at room temperature. Then, 10 ml of NALC-NaOH solution containing 2.9% natrium-citrat-dihydrat and 0.5% N-acetyl-L-cystein was added, and the sample was agitated for 25 min at 300 rpm in a shaker. After addition of 20 ml PBS, the sample was vortexed and centrifuged for 20 min at 3,800 × *g*. The supernatant was discarded, 10 ml PBS was added, and the sample was centrifuged as before. The supernatant was discarded, and the pellet was resuspended in 1 ml PBS and homogenized thoroughly by vortexing. Of the resuspended pellet, 200 μl was transferred to one slant of Löwenstein-Jensen medium with polymyxin B, amphotericin B, carbenicillin, trimethoprim (PACT), and glycerin, and to two slants of a Coletsos medium with PACT (both Artelt Enclit GmbH, Wyhra, Germany). Tubes were incubated for 1 week in horizontal position, afterward in upright position for up to 12 weeks. Cultures were examined every 2 weeks for colony growth. Once visible colonies appeared, presence of *M. bovis* was confirmed by real-time PCR targeting IS*1081* (44) and by endpoint PCR targeting RD4 (33). Nasal swabs and feces were examined in the same way.

#### Radiological Examination by Computed Tomography (CT) and Digital 2D Radiography (DR)

For CT scanning, lungs were placed into two plastic bags inside a plastic container. The lungs were scanned as described for trial 1, and data were analyzed with Synedra personal view^©^ and 3D Slicer^©^ (https://www.slicer.org/). Synedra was used to count lesions in the slices and record their size. Every slice was examined, and larger lesions were followed from beginning to end to avoid re-counting the same lesion in consecutive sections. Round and polygonal lesions could be distinguished. The largest diameter of each lesion was used as parameter for lesion size. The lesions were categorized into three classes: (I) small mineralized lesions <5 mm in diameter, (II) mineralized lesions more than 5 mm in diameter, and (III) partially mineralized confluent lesions >10 mm in diameter and containing several mineralized nuclei. The volume of the altered lung tissue was determined, both automatically and with the free hand tool of the 3D Slicer software. For automatic measurement, the threshold function was used with the range set from 250 to maximum Hounsfield units (HUs). Free-hand measurement was conducted for small or less mineralized lesions, which were not completely recognized by the threshold function. These lesions were marked with the free hand tool in every slice. The software allowed for adding up the lesion volume of the automatic and freehand measurements.

Later, digital 2D radiography images were taken of each lung using a Blueline-Ultra-1040HF x-ray apparatus (WDT) with Leonardo-Thales-DR-Kofferset wireless devices. The settings were 60 kV and 2.5 mAs exposure with a distance of 70 cm between the x-ray tube and the detector. The right and left lung were x-rayed separately in the dorsoventral and latero-lateral orientations. Four images per lung were analyzed with the Dicom PACS® software (Oehm and Rehbein, Rostock, Germany).

## Results

### Establishment of the Intrabronchial Application Procedure (Trial 1)

The anesthesia achieved with the protocol applied was found to be superficial, with the animals responding to external stimuli, but sufficiently deep to prevent defensive movements. The 85-cm working length of the bronchoscope allowed for access to all three sites selected for application. Correct positioning of the catheter for the applications was fast and easy to find with the help of the video image of the bronchoscope. The *bronchus (b.) tracheobronchialis*, which is the first diversion on the right side of the trachea, was used for orientation. The 1:2.5 dilution of barium sulfate suspension in water was easily applicable using a standard catheter and viscous enough to adhere to the mucosa, and 1-ml boli were deposited at the entrances to the *b. trachealis*, the right main bronchus, and the left main bronchus under visual control. Further distribution of the contrast medium could not be followed by bronchoscopy, because it completely filled the bronchial lumina and obscured the view.

**Figure 4:**
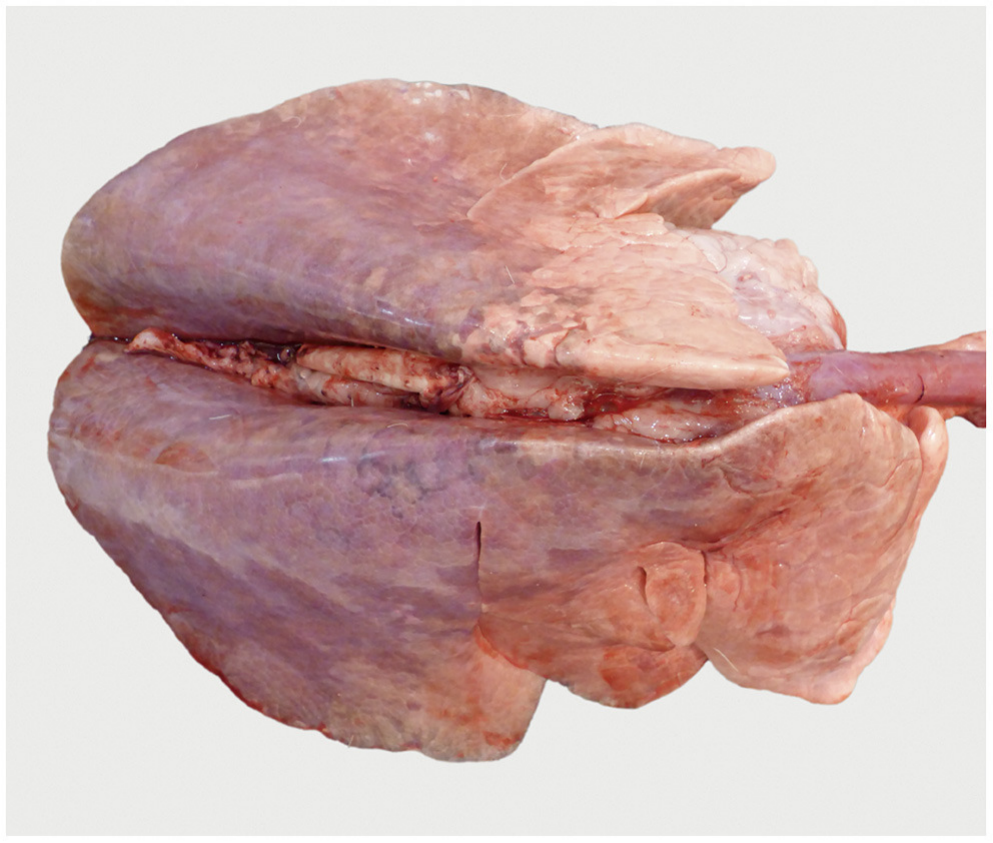
Lung after intratracheal fixation: voluminous, not collapsed parenchyma because of the instilled NBF. Dark red areas indicate retained blood, light areas indicate retained air. The distinctive distribution is caused by the position of the goats, lying on their back, during instillation of NBF.

**Figure 5:**
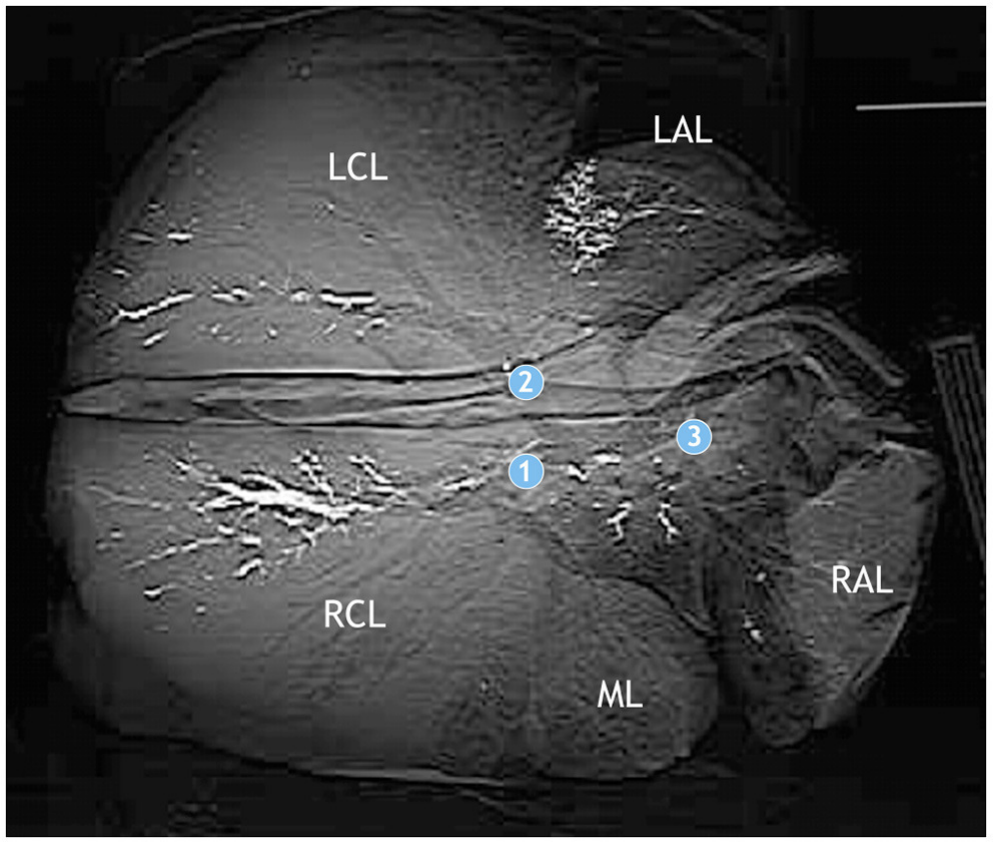
Computed tomography of the lung of a goat after application of barium sulfate suspension. Application sites are indicated by circles with numbers as in Figure 1. Barium sulfate (white) is present in the bronchial system of the left and right apical lobes (LAL, RAL), the accessory lobe, and the left and right caudal lobes (LCL, RCL), but not the middle lobe (ML). The amount varies among the lobes.

Examination of lungs following euthanasia of the goats and intratracheal fixation of the lungs 30 min after deposition of the contrast medium did not reveal macroscopic lesions (Figure 4). Subsequent CT scans showed contrast medium in left and right lung lobes and in the accessory lobe. It had accumulated around the three application sites and extended further down the respective airways (Figure 5). The amount of barium sulfate suspension was not evenly distributed among the sites.

### Inoculation of Goats With *M. bovis* and *intra vitam* Assessment (Trial 2)

Based on experiences from trial 1, the number of application sites was increased from three to five, adding *b. lobaris cranialis* and *b. lobaris medialis* as application sites for the *M. bovis* suspension (Figure 3). The increased number of inoculation sites was compensated for by reducing the volume of inoculum deposited at each site to 500 μl. The *M. bovis* inoculum was more liquid than the barium sulfate suspension allowing for the use of a spray catheter to achieve a more even distribution of the inoculum. All the five sites selected for inoculation could be reached with the bronchoscope, and correct positioning of the catheter was fast and easy under video guidance. The spray catheter allowed for the application of the inoculums at all sites from a single syringe, step-by-step, with little pressure.

The goats remained clinically healthy and in good general condition from the time of inoculation to the time of SICCT testing. They were blithely and intent, and had an undisturbed appetite and an average rectal temperature of 38.9 ± 0.3°C (mean ± SD of all daily measurements over time; *n* = 4 animals). Cultures of nasal swabs and fecal samples taken for mycobacterial growth on the day of inoculation and then daily from 1 dpi until 13 dpi and 21, 28, 56, 84, 112, and 140 dpi did not yield positive results throughout. All goats inoculated with *M. bovis* presented clearly positive SICCT 137 dpi, indicating a 100% infection rate. The control goats developed neither local nor systemic reactions in response to SICCT.

### Comparative Computed Tomography (CT) and 2D Digital Radiography (DR) Imaging of Lungs From *M. bovis*-Inoculated Goats

**Figure 6:**
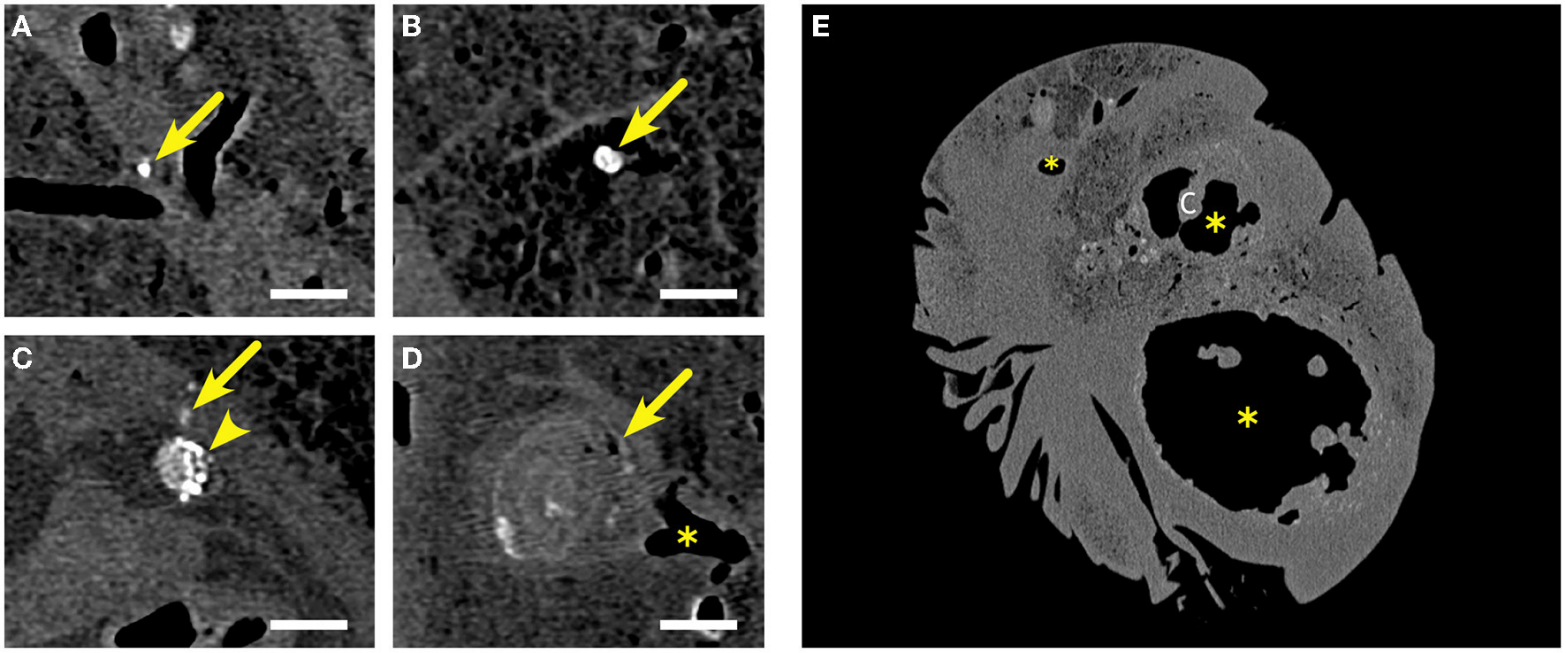
Examples of lesions detected by computed tomography in the fixed lungs of goats inoculated with *M. bovis*. **(A,B)** Small lesions <5 mm in diameter (arrows), mineralized (indicated by the white color) and with a density of >250 HU. **(C)** Lesion between 5 and 10 mm in diameter, mostly mineralized (arrowhead) next to a small lesion <5 mm diameter, mineralized (arrow). **(D)** Lesion exceeding 10 mm in diameter with multiple small foci of mineralization (arrow) located at a bronchial branching (^*^). **(E)** Several caverns (^*^) of variable sizes with highly variable outline and protruding *cristae* (c). Walls of the caverns are of variable thickness with multifocal mineralization (density >250 HU). Lumina are hypodense, indicating that they are filled with air. Caverns are surrounded by numerous mostly mineralized lesions of variable sizes. Bars in **(A–D)** = 10 mm.

CT allowed to identify the different lung lobes based on branching of the bronchial tree and to distinguish different types of lesions in the lungs of the goats inoculated with *M. bovis* (Figure 6; see Supplementary Table 1). Most lesions presented as small, round hyperdense foci with smooth borders. They had a density of more than 250 HU, indicating that they were mineralized. The diameter of these lesions was below 5 mm. The pulmonary tissue surrounding the mineralization did not present with increased density. The second category consisted of larger round or pleomorphic lesions with a diameter of more than 5 mm and significant variation in density. Most of the lesions were mineralized or partly mineralized. Sometimes, a fine dust of mineralization was present in the center. These lesions were surrounded by a tissue of higher density than the lung tissue. Lesions of the third category had a diameter of more than 1 cm. Some had multiple foci of mineralization and were of irregular shape. They were interpreted as result of confluent lesions. Most of the granulomas were located at the application sites of the inoculum and in proximity to bronchi. Frequency and distribution of the types of lesions varied among individual animals (Table 1), but small, mineralized lesions were detected most frequently. CT allowed to determine total numbers of lesions, total lung volume, total lesion volume, and, thus, percentage of lung volume affected by the lesions (Table 2). The percentage of affected lung volume was low in three animals, but reached 34% in one goat.

**Table 1:** Frequency and distribution of types of lesion detected by computed tomography (CT) in *Mycobacterium bovis*-inoculated goats.

**Location of lesions (lung lobe)**		**Lesions** ** <5 mm, mineralized**				**Lesions** **>5 mm, mineralized**				**Lesions** **>10 mm, irregular mineralization**			
		**Goat #418**	**Goat #423**	**Goat #428**	**Goat #436**	**Goat #418**	**Goat #423**	**Goat #428**	**Goat #436**	**Goat #418**	**Goat #423**	**Goat #428**	**Goat #436**
Right lung	*L. apicalis*	8	5		10	1	1		3		2		2
	*L. medialis*	1		1									
	*L. caudalis*	15	2	2	2	5			1	2			
	*L. access*.		27	2									1
Left lung	*L. apicalis*			4	17				1				
	*L. caudalis*	10			4	1		1		1		1	
Total number of lesions (by size)		34	34	9	33	7	1	1	5	3	2	1	3

**Table 2:** Number and volume of pulmonary lesions and percentage of affected lung tissue in *M. bovis*-inoculated goats.

	**Goat #418**	**Goat #423**	**Goat #428**	**Goat #436**
Lesions <5 mm	34	34	9	33
Lesions >5 mm	7	1	1	5
Lesions >10 mm	3	2	1	3
Caverns detected by CT	0	0	0	2
Total number of lesions	44	37	10	43
Total lung volume in cm^3^	2415.64	2499.78	2695.32	3255.08
Total lesion volume in cm^3^	9.78	33.68	7.61	1116.99
Lesion volume (%)	0.41	1.35	0.28	34.31

**Figure 7:**
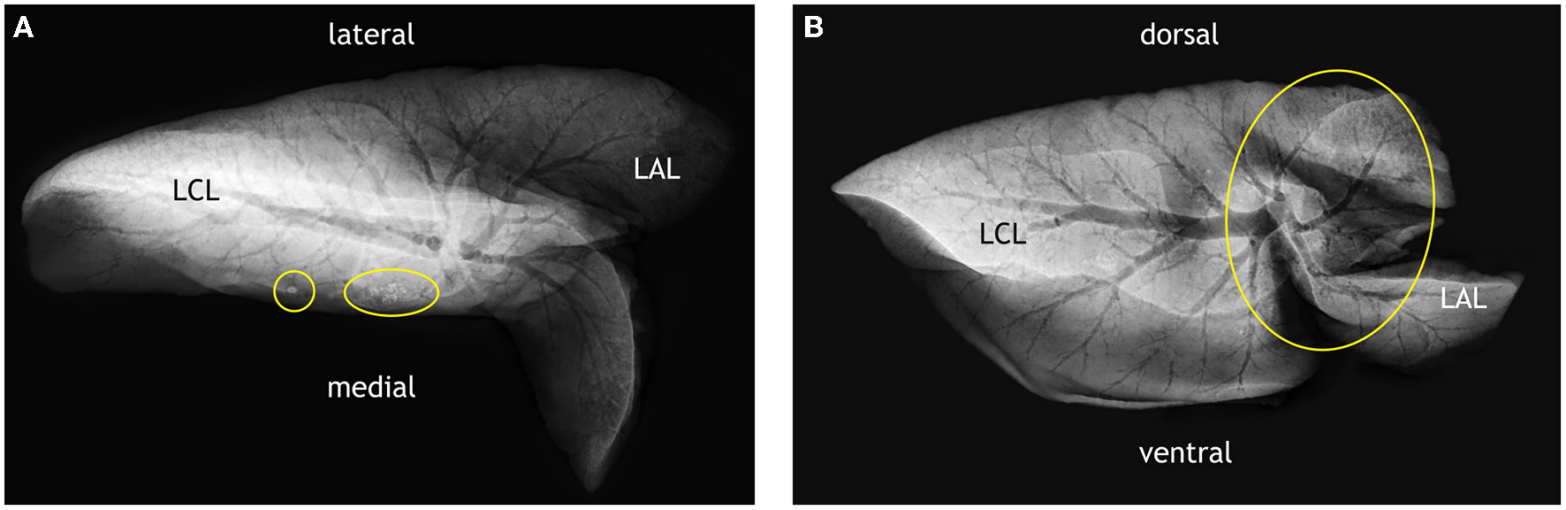
Examples of lesions detected by 2D radiography in the fixed lungs of goats inoculated with *M. bovis*. **(A)** Radiograph of the left lung of goat #428 in a dorsoventral view (lateral and medial sites indicated). Several mineralized and partly mineralized lesions (circles) are present in the left caudal lobe (LCL). **(B)** Radiograph of the left lung of goat #436 in a latero-lateral view (dorsal and ventral sites indicated). Caudal lung lobe (LCL) and apical lung lobe (LAL) are overlapping as indicated by the circle.

DR imaging gave a distinct outline of the lung tissue. The airways presented as hypodense structures. The identification of pulmonary lobes (left and right apical lobes, middle lobe, left and right caudal lobes, and accessory lobe) was based on their outline and branching of airways. The lungs of all the goats inoculated with *M. bovis* had tuberculous lesions, i.e., mineralized and partly mineralized foci of >1 cm size (Figure 7A). The cavernous lesion of goat #436 was clearly outlined by mineralization along the edges. Even though right and left lung lobes were dissected at the *bifurcatio tracheae* and, hence, separately radiographed, and images were taken in two orientations (dorsoventral and latero-lateral), overlapping of the pulmonary lobes hampered the identification of the exact location of lesions and assignment to lobes, as well as the determination of number and size of lesions (Figure 7B).

**Figure 8:**
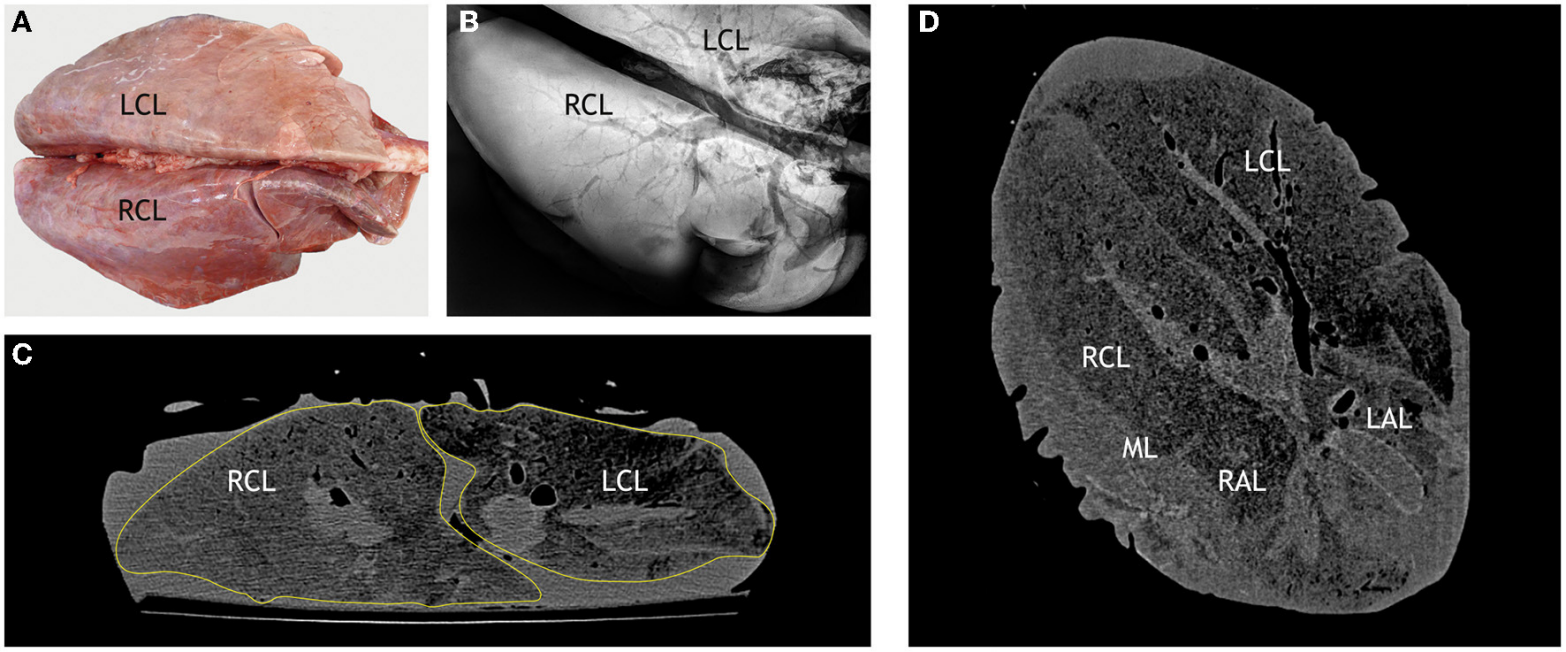
Correlation of macroscopic, radiographic, and computed tomography (CT) findings in a control goat. **(A)** Dorsal view of a lung without lesions after intratracheal instillation with NBF, right and left caudal lobes (RCL, LCL) are indicated. **(B)** Dorsoventral radiographic view of the same lung lobe: distinct outline of the homogenous pulmonary parenchyma (RCL, LCL indicated) and hypodense airways. **(C)** CT scan in transversal plane through the right and left caudal lobes (RCL, LCL). Pulmonary parenchyma is outlined by a yellow line. **(D)** CT scan in dorsal plane through the right and left caudal lobes (RCL, LCL), right and left apical lobes (RAL, LAL) and middle lobe (ML). Control goat #417 was inoculated with PBS.

Nevertheless, comparison of lesions detected by adspection, by CT, and by DR in the control goats and individual goats inoculated with *M. bovis* unveiled some degree of correlation. CT of the lungs of control goats that had no macroscopic lesions (Figure 8A) revealed homogeneous lung parenchyma with a density of below 250 HU (Figures 8C,D). There were no hyperdense areas indicative of pulmonary lesions. This was confirmed by the x-ray images (Figure 8B).

**Figure 9:**
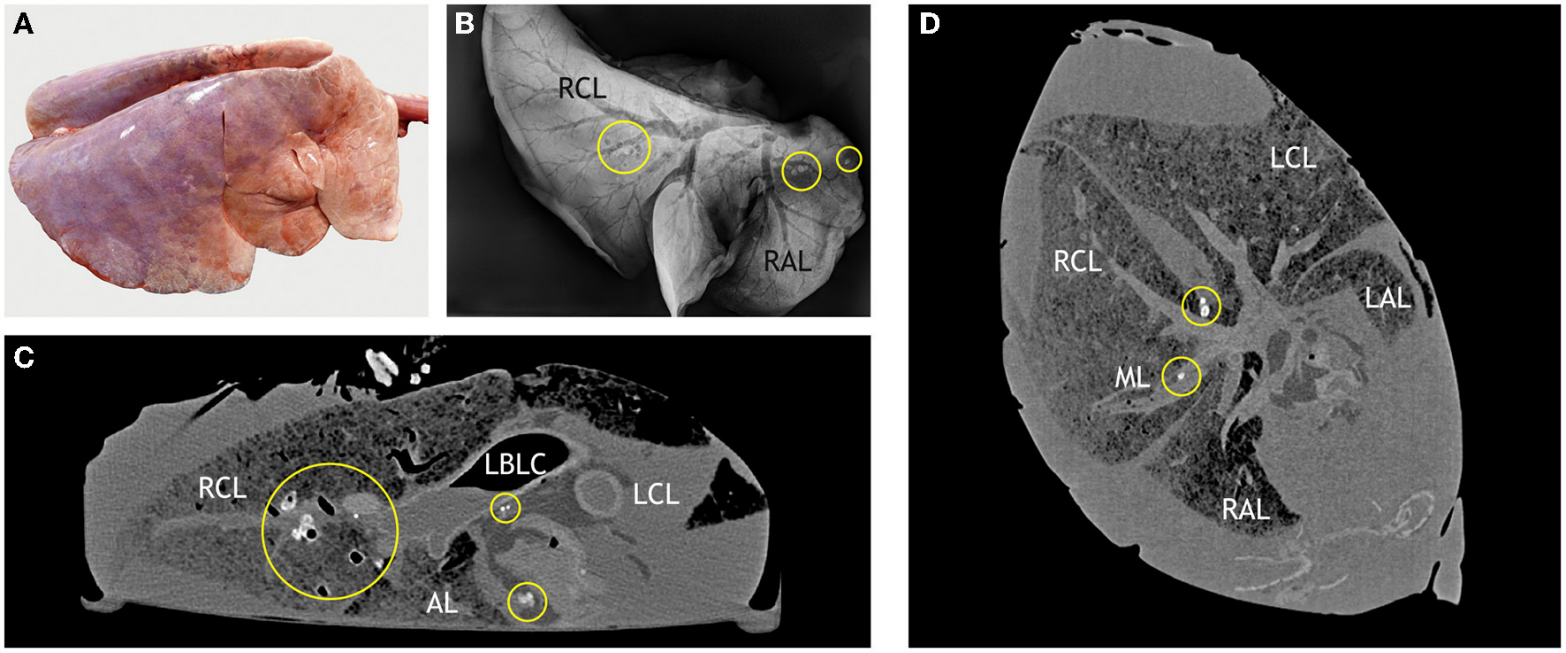
Correlation of macroscopic, radiographic, and computed tomography (CT) findings in a goat inoculated with *M. bovis* that developed no superficial lesions. **(A)** Macroscopic findings comparable to those in control goats (Figure 8A). **(B)** Dorsoventral radiographic view of the right lung reveals multiple mineralized lesions (indicated by circles) in the right caudal lobe (RCL) in close association to a ventral segmental bronchus (hypodense) and in the cranial part of the apical lobe (RAL) in close association to a segmental bronchus. **(C)** CT scan in transversal plane through the right and left caudal lobes (RCL, LCL) and accessory lobe (AL) shows mineralized lesions in the RCL and in the LCL (indicated by circles). The lesions are associated with the bronchial tree (left *bronchus lobaris caudalis*, LBLC, as example). **(D)** CT scan in dorsal plane through the right and left caudal lobes (RCL, LCL) and right and left apical lobes (RAL, LAL) shows lesions (indicated by circles) near the main bronchus of the RCL and one small lesion near the main bronchus of the medial lobe. Goat #418, inoculated with *M. bovis*.

The numerous lesions counted in goat #418 by CT were located deep within the parenchyma and not detected by adspection (Figure 9A). The lesions were predominantly <5 mm in diameter and mineralized (Figures 9C,D), but several lesions >5 mm in diameter and larger confluent lesions were also detectable. The majority of the lesions was located in close association to the bronchi, especially the ventral segmental bronchi of the right and left caudal lobes (Figure 9C). Some lesions were found in the apical lobe of the right lung. By radiography, only a few hyperdense lesions were detected in the left lung and in the caudal and accessory lobes of the right lung (Figure 9B). As observed by CT, they were located close to the bronchi. The numerous small lesions (<5 mm) revealed by CT could not be identified by DR.

In goat #423, predominantly small mineralized lesions <5 mm were detected by CT. They were most numerous in the right lung and the accessory lobe. Larger lesions were present in the right apical lobe. The left lung was free of lesions. Most of the lesions were located around the bronchi, but two lesions were close to the pleura. Corresponding round hyperdense structures were detected in DR radiographs of the right lung, although lesions in the cranial lung could not be assigned to distinct lobes.

In goat #428, the smallest number of lesions was detected by CT. Small mineralized lesions were rather evenly distributed in the middle, caudal, and accessory lobes of the right lung and in the apical and caudal lobe of the left lung. They were located near the bronchi, especially at their ventral aspect. A larger, irregular shaped hyperdense structure was present in the left caudal lobe 2 cm beneath the lung surface. DR radiographs revealed irregular hyperdense structures in the lobar bronchi of the bifurcation. Two hyperdense areas with irregular outline were present in the left caudal lobe close to the surface. The small lesions (<5 mm) revealed by CT could not be identified by DR.

**Figure 10:**
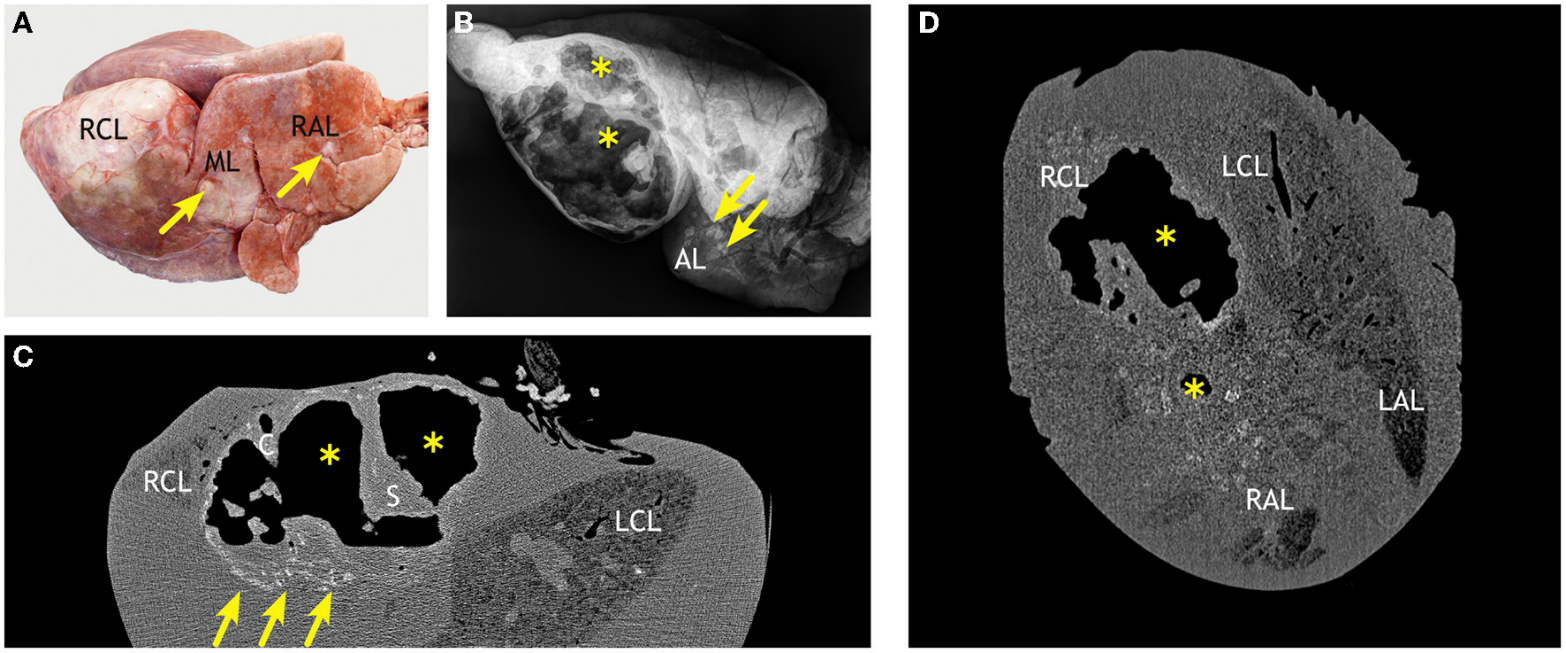
Correlation of macroscopic, radiographic, and computed tomography (CT) findings in a goat inoculated with *M. bovis* that developed severe lesions and extensive caverns. **(A)** Markedly enlarged right caudal lobe (RCL) and multiple white nodules in the middle (ML) and apical (RAL) lobes (arrows, examples). **(B)** Latero-lateral radiographic view of the right lung reveals large caverns (^*^) and mineralized lesions (arrows, examples) in the accessory lobe (AL). **(C)** CT scan in transversal plane shows that the air-filled caverns (^*^) with *septae* (S) and *cristae* (C) are predominantly located in the right caudal lobe (RCL) and cause its dorsal protrusion. Multiple mineralized lesions are prominent at the ventral aspect of the caverns (arrows). **(D)** The dorsal plane of CT scans through the caudal lobes (RCL, LCL) and apical lobes (RAL, LAL) confirm the irregular outline of the air-filled caverns (^*^) and reveal the extension of confluent partly mineralized lesions in the adjacent pulmonary parenchyma. Goat #436, inoculated with *M. bovis*.

Lesions were most extensive in goat #436 (Figure 10). Numerous small mineralized lesions were detected in the left and right apical lobes by CT. A large cavern extended throughout most of the right lung (Figures 10C,D). The cavern was outlined by an irregularly shaped, hyperdense wall which varied in thickness (up to 1 cm) and had multiple mineralized foci (>250 HU). Many hyperdense *cristae* and *septae* extended into the cavern and subdivided it. Its center was hypodense, indicating that it was filled with air. The cavern was surrounded by numerous mineralized lesions of variable size. Size difference between the left and right lungs was also evident by DR imaging. The right lung was distended by the large cavern with features comparable to those described by CT (Figure 10B). Distinct mineralized structures were present in the cranial part of the right lung without connection to the cavern.

### Pathological Findings in the Lungs of *M. bovis*-Inoculated Goats

Gross examination was initially limited to external inspection to keep the lungs intact for subsequent CT and DR examination. Macroscopic lesions were detected in none of the controls and only in the lungs of one of the goats inoculated with *M. bovis* (goat #436). Here, the lesions presented as chronic fibrous pleuritis of the right basal lobe with a circumscribed adhesion between the visceral and parietal serosa. The lung was asymmetric with a markedly enlarged right caudal lobe (Figure 10A). Multiple white to yellow nodules, 1–15 cm in diameter, were irregularly distributed throughout the right apical, middle, and caudal lobes.

**Figure 11:**
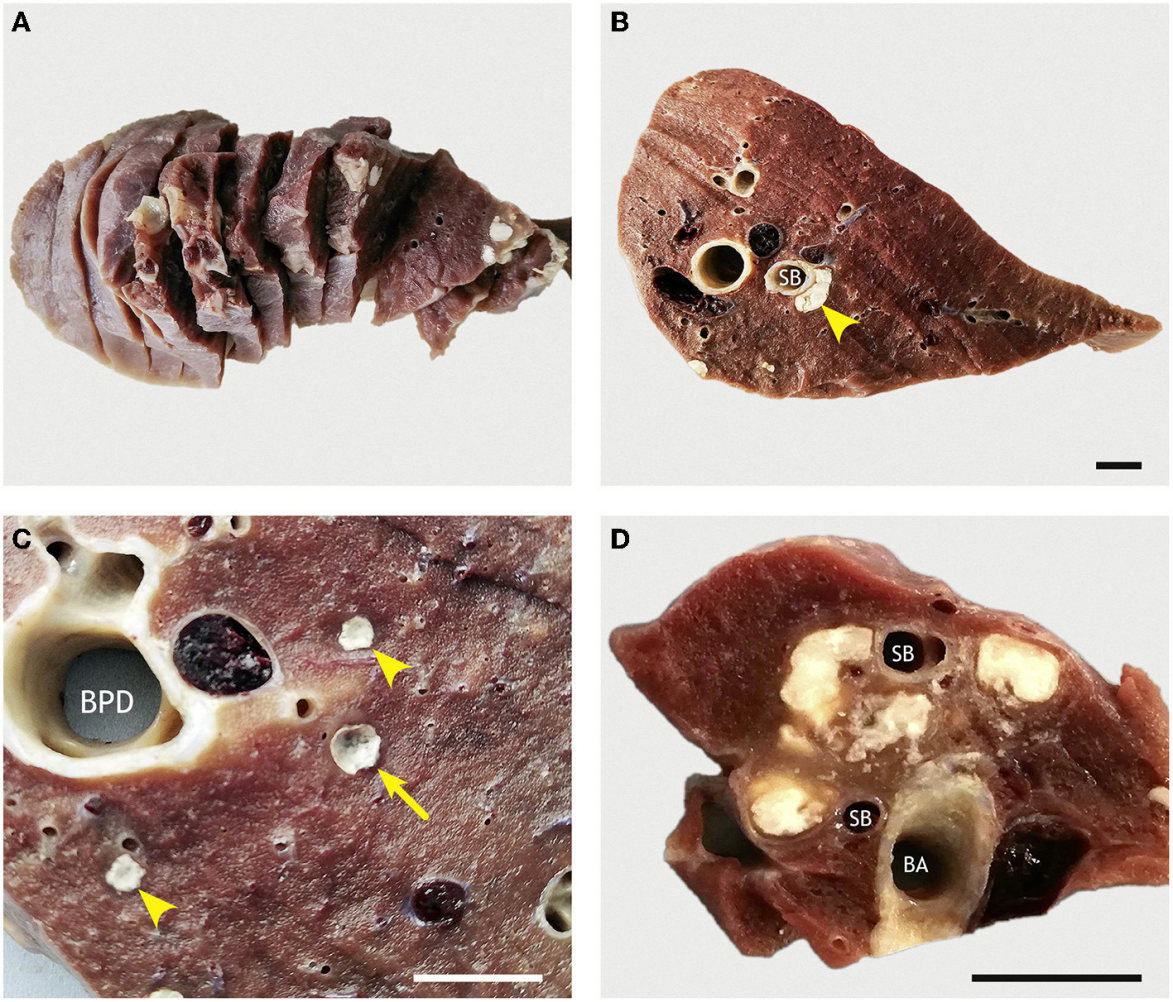
Sliced lung of a goat inoculated with *M. bovis* that developed no superficial lesions. **(A)** Pulmonary lobes (accessory lobe) were completely sliced at 1-cm thickness. **(B)** Section of the RCL with confluent granulomas (arrowhead) surrounding the ventral aspect of a ventral segmental bronchus (SB). **(C)** Higher magnification of pulmonary tissue next to the *bronchus principalis dexter* (BPD) reveals two small caseous granulomas (arrowheads) and one small cavern (arrow). **(D)** Multiple confluent caseous granulomas located between *bronchus accessorius* (BA) and branching segmental bronchi (SB) in the accessory lobe. Goat #418, inoculated with *M. bovis*. Bars = 1 cm.

**Figure 12:**
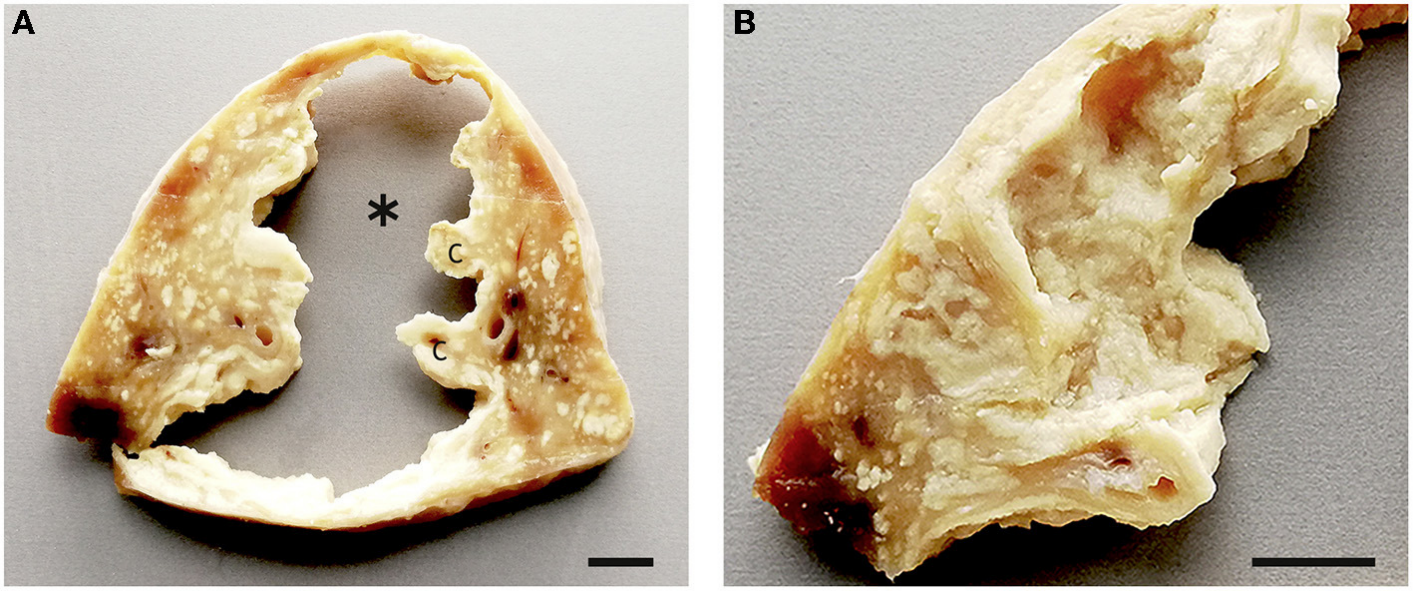
Sliced lung of a goat inoculated with *M. bovis* that developed severe lesions and extensive caverns. **(A)** Section through the extensive polygonal cavern (^*^) with multiple *cristae* (C) in the RCL. The surrounding pulmonary tissue is replaced by confluent caseous granulomas with multiple foci of mineralization. **(B)** Higher magnification of the highly corrugated lining of the cavern (^*^). Goat #436, inoculated with *M. bovis*. Bars = 1 cm.

Based on CT findings, the lungs of one goat without superficial macroscopic lesions (goat #418, Figure 11) and of the goat with lesions (goat #436) were dissected (Figure 12). Macroscopic examination of the dissected lung without superficial lesions revealed nine caseous granulomas (1–4 mm in diameter) in the proximal half of the right apical lobe. In the right caudal lobe, multiple granulomas (2–11 mm in diameter) and caverns (2–13 mm in diameter) were located proximal to the ventral segmental bronchi and smaller medio-ventral airways. Larger confluent granulomas were located at the ventral aspect of the bronchi (Figure 11B). The granulomas were filled with a white caseous material (Figure 11C). The caverns had empty centers surrounded by irregularly thickened walls of the white caseous material. In the accessory lobe, multiple confluent granulomas (10 × 10 × 16–30 mm^3^) were present in the proximal part of the branching segmental bronchi (Figure 11D), and there was a medium-sized cavern (10 × 16 × 5 mm^3^).

The macroscopic examination of the dissected lung with superficial lesions (goat #436) revealed that most of the pulmonary tissue was replaced by a large polygonal cavern of ~70 mm diameter. The cavern had a highly irregular outline and multiple protruding *cristae* (Figure 12), and was surrounded by confluent granulomas with discrete foci of caseous necrosis.

**Figure 13:**
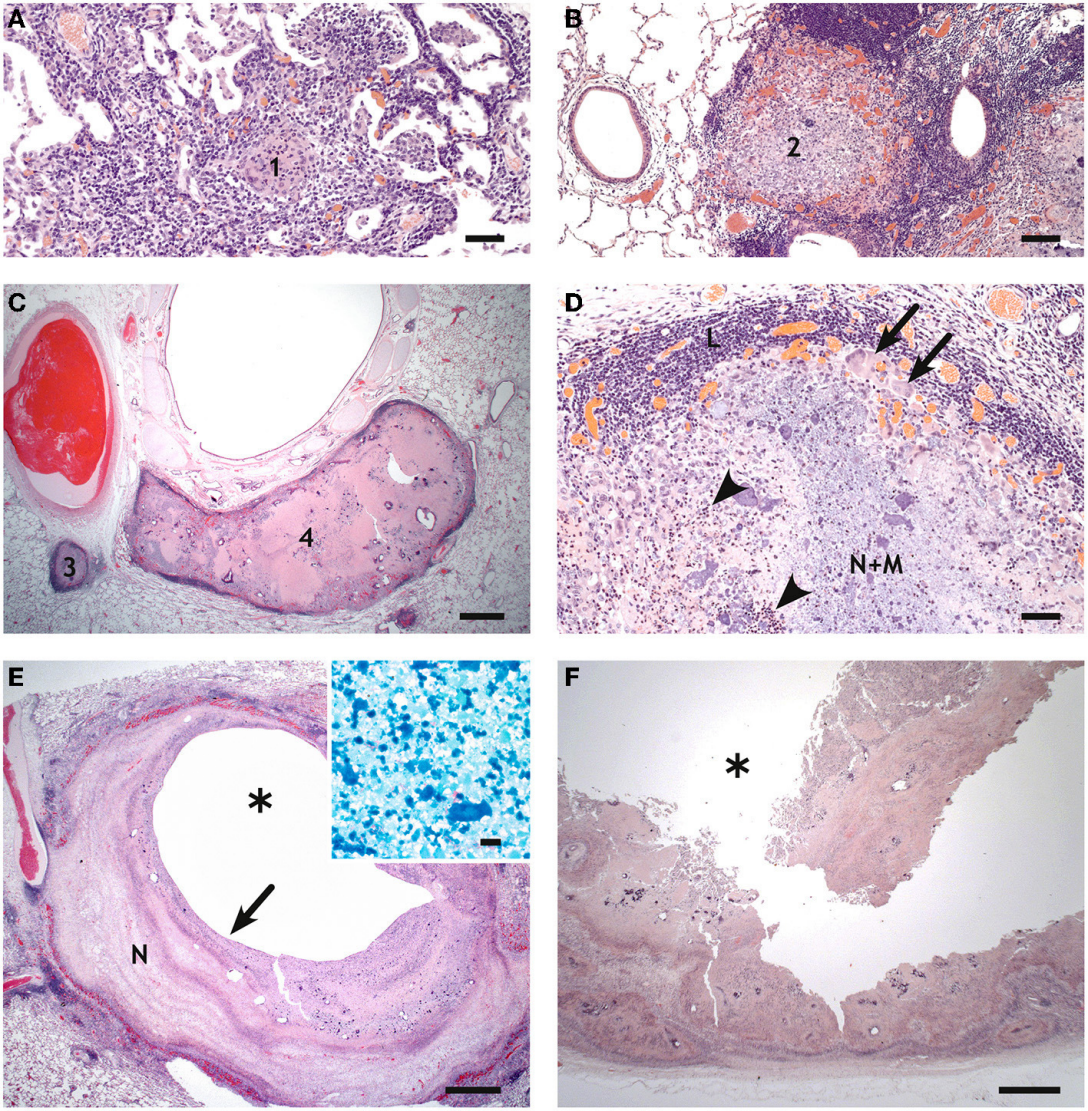
Histology of pulmonary lesions in goats inoculated with *M. bovis*. **(A)** Type 1 granuloma (1) consisting of epitheloid cells in the accessory lobe of goat #418, bar = 50 μm. **(B)** Type 2 granuloma (2) with predominantly epitheloid cells and minimal necrosis in the apical lobe of goat #418, bar = 100 μm. **(C)** Type 3 (3) and type 4 (4) granulomas in the caudal lobe of goat #418, bar = 1,000 μm. **(D)** Higher magnification of the type 3 granuloma in **(C)** shows central necrosis and mineralization (N+M), neutrophils (arrowheads, examples), epithelioid cells and multinucleated giant cells (arrows, examples), and a lymphocytic infiltrate (L), bar = 50 μm. **(E)** Small cavern (^*^) in the caudal lobe of goat #418. The central cavity is surrounded by multiple layers of necrosis (N), bar = 1,000 μm; inset: a few AFBs in the necrotic wall (site indicated by arrow), bar = 10 μm. **(F)** Highly corrugated wall of the large cavern (^*^) of goat #436. The pulmonary tissue is replaced by confluent granulomas. Bar = 1,000 μm.

By histology, small granulomas in both lungs were classified as type 1, 2, or 3, larger granulomas as type 3 or 4, sometimes with type 1 satellite granulomas (Figures 13A–D). The granulomas were not well-demarcated, and inflammatory cells including epitheloid cells were present in surrounding alveoli. The caverns resembled the granulomas, with most of the central necrotic material missing (Figures 13E,F). A part of the wall was lined by bronchial epithelium in some of the caverns. No AFB was present in any of the granulomas, but a few (++) AFBs were present in the necrotic material around one of the smaller caverns (Figure 13E inset).

### Pathological Findings and Cultural Isolation of *M. bovis* From Other Tissues of *M. bovis*-Inoculated Goats

All the inoculated goats, but none of the controls, presented granulomas in the LNN *tracheobronchiales* or LNN *mediastinales* (see Supplementary Table 2). In three goats, macroscopic lesions presented as multiple, caseous granulomas. Histologically, all types of granulomas were seen, but types 3 and 4 granulomas were more frequent. AFBs were not detected in any of these granulomas. In the goat with severe pulmonary lesions (goat #436), LNN *tracheobronchiales* and LNN *mediastinales* were almost completely replaced by confluent caseous granulomas. Histologically, these were categorized as multicentric type 4 granulomas with satellite granulomas in the periphery. Single AFBs were present in a few multinucleated giant cells. In goat #436, type 1 granulomas were detected in the mucosa-associated lymphoid tissue at the ileocecal entrance and types 1, 2, and 3 granulomas in the LNN *mesenteriales* and *ileocolici*. These granulomas were negative for AFBs.

The *M. bovis*-inoculated goats had severe reactions characterized by necrotizing vasculitis, lymphohistiocytic perivasculitis, microabscesses, and severe edema in the dermis and subcutis at the site where bPPD had been injected. Alterations were associated with swelling, edema, and activation of the draining LNN *cervicalis superficialis*. The reactions at the injection site of aPPD were mild. No reactions were observed in the mock-inoculated control goats.

In tissues collected at necropsy, mycobacterial growth was detected in one or more tissues of all the goats inoculated with *M. bovis*, but none in those of the mock-inoculated control animals (see Supplementary Table 2). All positive cultures were confirmed to be *M. bovis. M. caprae* was not identified.

## Discussion

Compared to cattle, the main natural host of *M. bovis*, goats qualify as experimental animal species, particularly for infection experiments under high containment conditions, because of lower maintenance cost and easier handling by experimenters. The size of most goat breeds is favorable for the application of different imaging techniques to live animals or extirpated organs, and handling of the organs (fixation, transport, scanning) is easier than in cattle. Notably, goats are also natural hosts for members of the *M.tuberculosis* complex (MTC), namely, *M. bovis, M. caprae*, and *M. tuberculosis* (45). The combination of these factors makes these animals highly suitable models for TB research, for studying both the disease and effects of new vaccines (13, 45).

Different routes of infection have been successfully applied, but pathomorphological manifestations varied with inoculation procedure (13, 15, 21–23). This is particularly critical when minute differences in the virulence of bacterial strains, in the efficacy of vaccine candidates, or in the individual susceptibility of animals to tuberculosis morbidity and mortality shall be unveiled (46). As all inoculation procedures have their advantages and disadvantages, this study assessed a novel inoculation technique based on endobronchial administration but replacing the classical bolus application at the *bifurcatio tracheae* with a visually controlled application of a spray. The results of the different microbiological, immunological, pathological, and imaging methods indicate that this inoculation protocol produced disease in all inoculated animals and is at least equivalent to published protocols.

Experimental infection of cattle with *M. tuberculosis* isolates documents the attenuation of the human tubercle bacillus for cattle (47). Goats challenged by the transthoracic route with different members of the MTC displayed different clinical signs (45). Highest lesion scores were observed in *M. bovis*-challenged goats, followed by those challenged with *M. caprae*. Differences in the proportion of animals with viable bacteria in tissues and in response to the intradermal and gamma-interferon (IFN-γ) tests imply that *M. bovis* and *M. caprae* differ in their virulence and capability of activating host response (45). Even though *M. caprae* dominates endemic bTB infections in the alpine region in Europe affecting cattle and red deer (48), *M. caprae* is mostly recognized as a goat pathogen. Nevertheless, given the recognized heterogeneity of *M. caprae* strains circulating in animal populations (49, 50), we considered it favorable to use an *M. bovis* isolate as inoculum for the improvement of the caprine TB infection model. Once established, it would be highly intriguing to apply the model to unveil differences in the degree of virulence and host adaptation of *M. caprae* isolates.

It is generally accepted that, with some exceptions, cattle become infected with *M. bovis* by either the oral or the respiratory route (51). The oral route is most important in calves nursed by tuberculous cows, while the respiratory route is most common in adult cattle and facilitated by the animals' natural behavior. Using the oral, subcutaneous, or intravenous route of infection for experimental inoculation requires higher doses, lesions are frequently atypical, and animals show systemic reactions (52). Like in experimental TB infection of cattle (53–55), studies on goats have mostly mimicked the respiratory route by intratracheal, transthoracic, and endobronchial inoculation. Low doses (10^2^-10^3^cfu) of *M. bovis* or *M. caprae* reportedly resulted in 100% infection rates with lesions in the lower respiratory tract and systemic dissemination (13, 15, 22, 23). A caprine aerosol *M. bovis* infection model required high inoculation doses and did not result in the formation of cavernous lung lesions (21). Non-quantifiable, partial losses of the inoculum due to adherence to the surface of the endotracheal tube used for delivery of aerosolized bacteria, as well as due to the HEPA filter indispensable for clearing exhaled air, likely account for these deviations. Indeed, studies with *Burkholderia pseudomallei* indicated that only an estimated 10% of nebulized bacteria were deposited in the lungs (56). Applying for the first time a combination of video-guided bronchoscopy and spray application of an inoculum to several defined sites, we demonstrate that intrabronchial inoculation can be achieved with minimal technical effort in a more natural manner than bolus application.

All the goats inoculated in this study with a minute dose of *M. bovis* (4.7 × 10^2^ cfu) became infected, as deduced from the positive IGRA results and eventually confirmed by re-isolation of the inoculated strain from the mediastinal lymph nodes of all the inoculated goats. Installing the inoculum as spray, the liquid volume administered to a given area of the mucosal surface will be comparably higher than after aerosol inoculation, particularly as we flushed the catheter with 1 ml NaCl after installation of the inoculum. Physiological mechanisms comprising a retrograde movement of mucus by ciliated epithelia of airways and coughing may theoretically lead to removal of a part of the inoculum before *M. bovis* can enter cells and tissues on site. However, repeated sampling on the days after inoculation of nasal secretion and feces of the goats yielded negative results throughout. This strongly indicates that the effective infectious dose closely corresponds to bacterial titers in the inoculum. This finding is in positive contrast to studies deploying aerosol and seeder animal approaches that resulted in low estimated portions of the inoculum deposited in the respiratory tract and an incomplete infection rate of co-housed goats, respectively (21).

Aerogenic infection with *M. bovis* in goats resulted in lesions to similar those described for pulmonary disease in natural caprine TB (15). Researchers conducting endobronchial inoculation at the *bifurcatio tracheae* of goats in the right decubitus position experienced that the intrapulmonary extension of lesions to one or more lobes and, subsequently, the spread of mycobacteria to the draining lymph nodes was strongly influenced by the inoculation procedure (13). In our study, sedated animals were placed in sternal position for inoculation, which placed the thorax in a position comparable to an animal standing upright. With endoscopic-guided inoculation of the goats and deposition of several small aliquots of the inoculum, we achieved a wider and more even distribution than inoculation without a visible control (13, 45). The application of a contrast medium in a pre-study (trial 1) indicated that the procedure led to the inclusion of major sections of the bronchial tree, even when the medium was applied with a regular rather than a spray catheter and by approaching only three sites of the bronchial tree.

Endoscopy is instrumental for purposeful placing of inocula at pre-defined sites of the airways but requires special handling of animals, which poses a problem under high containment conditions. To ensure that the animals tolerate the procedure without defensive movements, the goats were subjected to anesthesia with midazolam and ketamine. Using midazolam instead of the commonly used xylazine led to reduced salivation and improved the bronchoscopic view (data not shown). The goats tolerated this procedure well, even though some of them needed a prolonged time for regaining consciousness. Other anesthesia protocols may be even more convenient (57). Alternatively, pushing the catheter *via* the nasal route could be applied with sedation and good fixation only, if the experimental animals are of appropriate size or age (58).

Application of CT scanning and analysis has been introduced to TB research on a goat model by de Val Perez et al. (13). In their protocol, the lungs and heart are removed at necropsy, lung gross lesions were recorded first by palpation of the different lobes, and then the whole lungs are fixed with formalin by pouring the fixative into the trachea while holding the lungs in a vertical position until the trachea is filled with fixative. The authors stressed the importance of instillation of the lungs with formalin to distend the lungs to approximately the same volume they would have in the pulmonary cavity. This renders the ratio of the lesion volume to the total lung volume comparable between different experiments and research groups. The use of this ratio also corrects for slight differences that could exist in sizes of animals and lungs, even in age-matched animals. Taken this recommendation into account, the protocol was modified in such a way that the lungs were instilled with a fixative solution before opening the thoracic cavity and consequently without squeezing the tissue by palpation to retain the original shape and size of the lung lobes as much as possible.

CT scanning is a highly sensitive and quantitative method for evaluation of pulmonary lesions, which allows to detect TB lesions <1 mm^3^ that would be difficult to find by direct macroscopic evaluation (35). To obtain a reasonable resolution of CT images, we faced the issue of density of the formalin solution. The lungs had to be immersed in formalin for >2 months for biosafety reasons before they could be transferred to the CT device located outside the high containment facility. The software deployed for the image analysis insufficiently differentiated the margin of the lungs because the attenuation value of formalin was very similar to the attenuation of the lung tissue. Therefore, the formalin solution was drained from the lungs as much as possible. In some cases, it was still unavoidable to mark the volume of the lung tissue on the digital image by hand. Although this was time-intensive, it guaranteed a detailed analysis of every virtual slice and, consequently, of every part of the lungs.

Perez de Val et al. (35) distinguished four different kinds of lesions (calcified, cavitary, solid, and complex) by CT and calculated the total pulmonary volume and the volume of lesions. Solid lesions with less calcification or caverns are difficult to detect automatically with the analysis software, and it is necessary to observe every slice to characterize and measure the TB lesions. While this is tedious, it bears the capacity to detect small changes in density patterns due to inflammatory reactions around granulomas that may not be visible by direct macroscopic observation. Granulomas classified as multicentric by histology often comprise multiple foci of mineralization. Some of them may have been identified as separate small granulomas <5 mm by CT. Multicentric granulomas could result from fusion of monocentric granulomas that have developed in close proximity. It was difficult to decide by CT whether some granulomas around the bronchial system and deeper in the lung tissue that were located very close to each other represented separate granulomas or one large confluent lesion. Comparison of CT and histologic findings of the same area, however, revealed that the granulomas were mostly separate entities.

The use of CT has several advantages compared to radiography. A three-dimensional view that can be reconstructed *in silico* by employing different programs for analyses allows for a more detailed evaluation of TB lesions. However, CT instruments are rarely available in high-containment facilities. Scanning of extirpated and fixated organs is a suitable workaround but only applicable for end-point determinations, as this depends on sacrifice of experimental animals. In contrast, digital radiography devices are rather small in size and weight, have comparably low investment needs, and come with surfaces that, at least in part, can be subjected to appropriate disinfection procedures. Thus, this technology offers an excellent opportunity to monitor the development of TB organ lesions *intra vitam* and over time. Therefore, we evaluated the applicability and resolution of a digital and portable radiography system for the infection model in goats. To mitigate an overlap of the lung tissue, which impedes the exact evaluation of lung lesions, especially regarding numbers and sizes, and to detect less mineralized granulomas, the left and right lungs were imaged separately. However, even this measure was insufficient to prevent a significant overlap. Furthermore, it was difficult to place the lungs always in the same position to allow for standardized evaluation. These problems do t arise in living animals. Therefore, digital radiography is an intriguing option to identify animals that develop lesions after inoculation and follow the development of TB lung lesions *intra vitam*.

Cavitary lesions are common features in advanced stages of human and caprine TB. Despite the severe necrosis found in some lesions, cavitary lesions were not detected in studies deploying aerosol inoculation (21). The authors speculated that, because of the progressive worsening of the animals' condition, they had to be sacrificed very early at 12 weeks p.i. Other studies that have applied the inoculum endobronchially in goats (13, 35) left the animals for 14 weeks and observed large cavitary lesions in the majority of the animals by then. In trials carried out on other species like badgers (59) and calves (60), using the endobronchial route of infection, lesions progressed slowly, resembling what is observed in natural cases of TB in these species (13). Particularly in low challenge-dose experiments in calves, large coalescent lesions were usually not found. Goats seem to show a faster progression of lesions, which can be considered advantageous for experimental conditions (13). We necropsied animals 20 weeks pi. Given the sizes of the lesions observed including a huge cavern in one animal, it is likely that, similar to the endobronchial application (13), the spray catheter application results in cavernous lesions early on.

## Data Availability Statement

The raw data supporting the conclusions of this article will be made available by the authors, without undue reservation.

## Ethics Statement

The animal study was reviewed and approved by Committee on the Ethics of Animal Experiments of the State of Thuringia, Germany; Committee on the Ethics of Animal Experiments of the State of Mecklenburg-Western Pomerania, Germany. Written informed consent was obtained from the owners for the participation of their animals in this study.

## Author Contributions

SK, LG, and CM coordinated the research project. HK conceived the study and planned and supervised the animal experiment, bacterial culture, and immunological experiments. EL-T and RU performed the necropsies. EL-T conducted macroscopic and histologic assessments of the lesions. SB supervised the molecular typing of the inoculum. NW, JF, and MR performed the animal experiments and analyzed the data. KP and NW analyzed the radiological data. SP made the radiographs. NW, CM, and EL-T drafted the manuscript. All the authors discussed the results, commented on the manuscript, and read and approved the final version of the manuscript.

## Funding

The project Innovative Vaccines against *Mycobacterium tuberculosis* complex (MTC) and *Staphylococcus aureus* for application in veterinary and human medicine (funding code 03ZZ0806C) was funded by the German Ministry for Education and Research (BMBF) as part of the consortium InfectControl 2020–Novel Anti-Infective Strategies.

## Conflict of Interest

LG was employed by Vakzine Projekt Management GmbH. The remaining authors declare that the research was conducted in the absence of any commercial or financial relationships that could be construed as a potential conflict of interest.

## Publisher's Note

All claims expressed in this article are solely those of the authors and do not necessarily represent those of their affiliated organizations, or those of the publisher, the editors and the reviewers. Any product that may be evaluated in this article, or claim that may be made by its manufacturer, is not guaranteed or endorsed by the publisher.
