# Supplementary material for: Video Endoscopy-Guided Intrabronchial Spray Inoculation of Mycobacterium bovis in Goats and Comparative Assessment of Lung Lesions With Various Imaging Methods
Source: Front Vet Sci. 2022 May 3;9:877322. doi: 10.3389/fvets.2022.877322 (PMC9113525; doi:10.3389/fvets.2022.877322)
Supplement: Supplementary file 1 [file Data_Sheet_1.pdf]

Supplementary Material

Supplementary Table 1. Histopathological characterization of selected granulomas found in the lungs of *M. bovis*-inoculated goats and comparison with computed tomography (CT) findings

type	lesions stage[1]	cellular composition[1]	necrosis and mineralization[1]	fibrosis, capsule[1]	CT lesion types[2]
1	initial	epitheloid macro-phages, ± giant cells, few lymphocytes, single PMN	no necrosis	-	undetectable due to missing/ minimal mineralization
2	solid	epitheloid macro-phages, ± giant cells, interspersed with lymphocytes and PMN	minimal necrosis and/or mineralization	incomplete, thin	
3	monocentric necrosis and mineralization	± epitheloid macro-phages, giant cells, lymphocytes and PMN, surround necrosis and/or mineralization	monocentric caseous necrosis and/or mineralization	variable - / +++	small or large lesions, mineralized or partly mineralized (I, II)
4	multicentric necrosis and mineralization	± epitheloid macro-phages, giant cells, lymphocytes and PMN, surround necrosis and/or mineralization	multicentric caseous necrosis and/or mineralization	variable - / +++	large, confluent lesions, mineralized (III)

- not present, + mild ++ moderate, +++ severe, ± present at variable numbers, PMN neutrophils
[1]characteristics of granulomas according to Wangoo *et al.*, 2005, modified; [2]this study

Supplementary Table 2. Granulomas and/or cultural isolation of *M. bovis* from organ tissues of *M. bovis*-inoculated goats sampled at necropsy

Organs	goat #418		goat #423		goat #428		goat #436	
	granuloma	culture	granuloma	culture	granuloma	culture	granuloma	culture
Tonsil	-	-	-	-	-	-	-	+
LN retropharyng. med.	-	-	-	-	-	-	-	+
LN tracheobronchialis sinister	+	-	+	+	+	+	+	+
LNN mediastinales	+	+	+	+	+	+	+	+
ileal Peyer's patch	-	-	-	-	-	-	(+)[1]	+
LNN ileocolici	-	-	-	+	-	-	+	+
LNN mesenteriales	-	-	-	-	-	-	+	+

- negative for *M. bovis*/no granuloma; + positive for *M. bovis*/granuloma present; [1]granuloma present in mucosa-associated lymphoid issue at the ileocecal entrance, not in the ileal Peyer´s patches
